# Supplementary material for: One-Step Construction of Tryptophan-Derived Small Molecule Hydrogels for Antibacterial Materials
Source: Molecules. 2023 Apr 10;28(8):3334. doi: 10.3390/molecules28083334 (PMC10141015; doi:10.3390/molecules28083334)
Supplement: Supplementary file 1 [file molecules-28-03334-s001.zip › molecules-2269413-supplementary.pdf]

molecules

Supplementary Materials

One-Step Construction of Tryptophan-Derived Small Molecule Hydrogels for Antibacterial Materials

Xianwen Song †, Shunmei He †, Jun Zheng, Shutong Yang, Qiang Li and Yi Zhang *

Hunan Provincial Key Laboratory of Micro & Nano Materials Interface Science, College of Chemistry and Chemical Engineering, Central South University, Changsha 410083, China; songxianwen.sxw@foxmail.com (X.S.); h1757234106e@163.com (S.H.); zhengjun@csu.edu.cn (J.Z.); yangst@csu.edu.cn (S.Y.); liqiang0410@outlook.com (Q.L.)

* Correspondence: yzhangcsu@csu.edu.cn

† These authors contributed equally to this work.

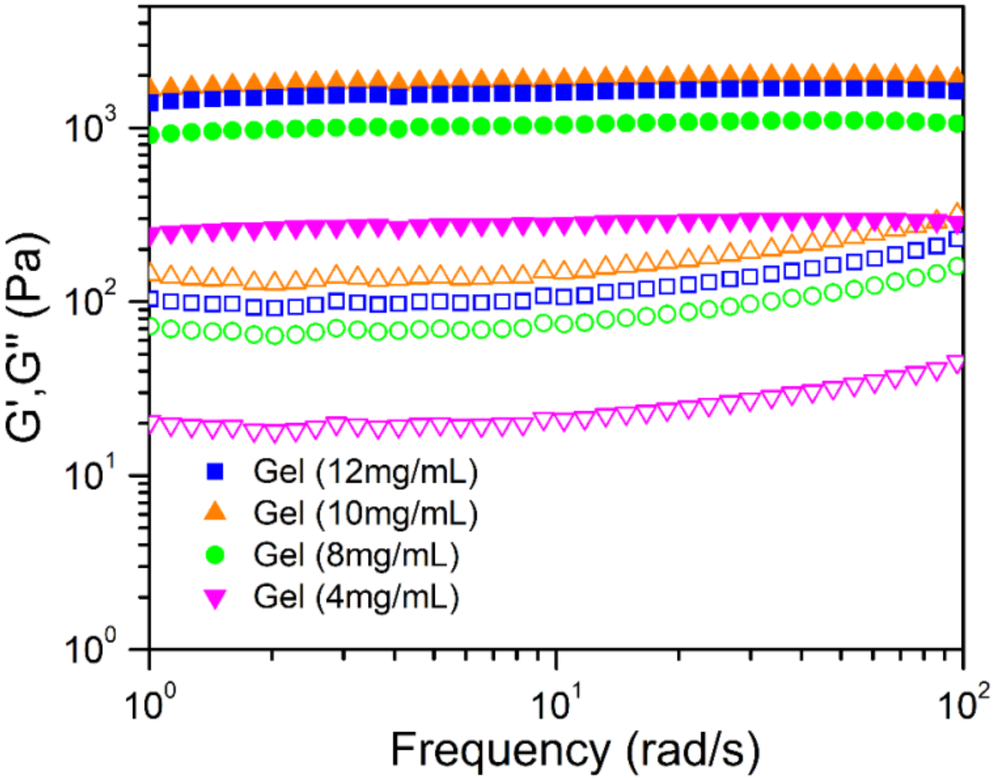

Figure S1. Frequency sweep of the ZW/GDL hydrogels with different concentrations.

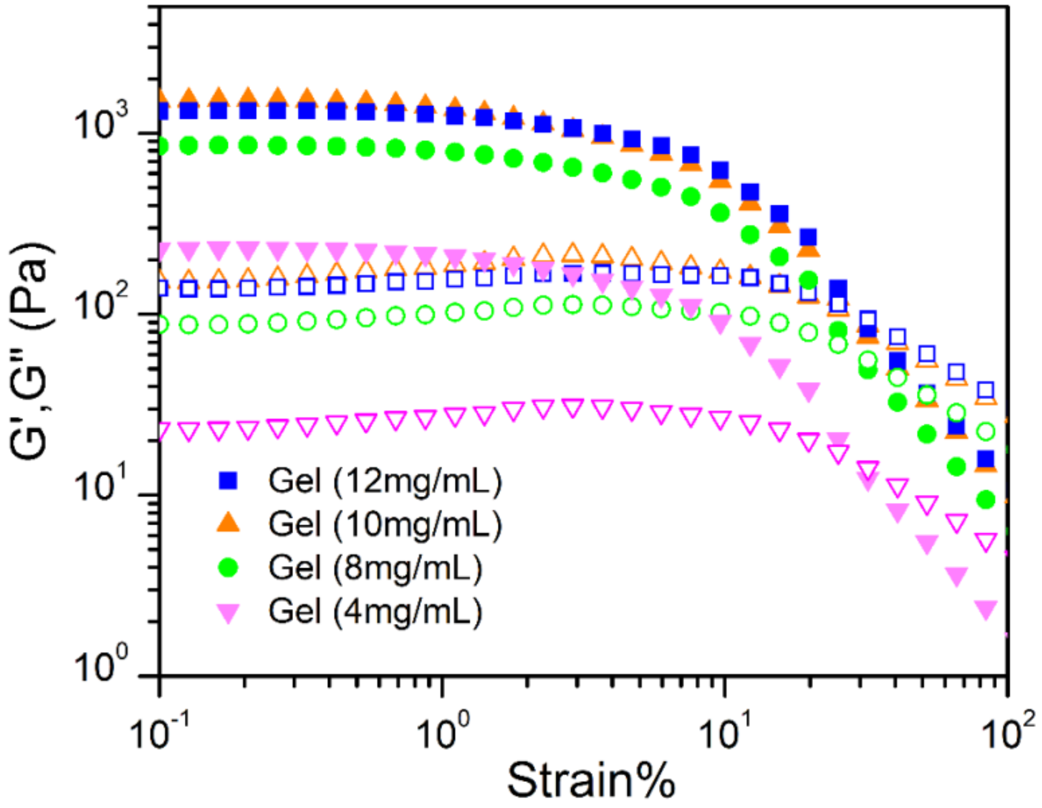

Figure S2. Dynamic oscillatory stress sweep of the ZW/GDL hydrogels with different concentrations.

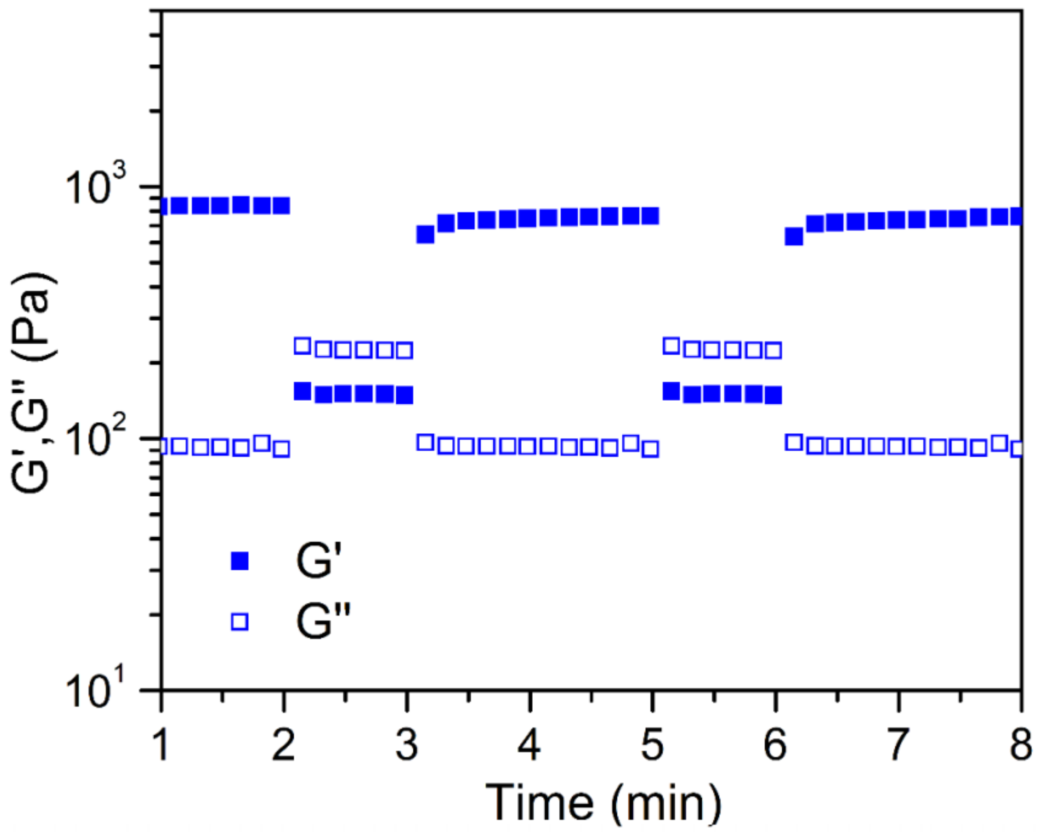

Figure S3. Alternate Step-strain sweep measurements of the ZW/GDL hydrogels at 6 mg/mL.

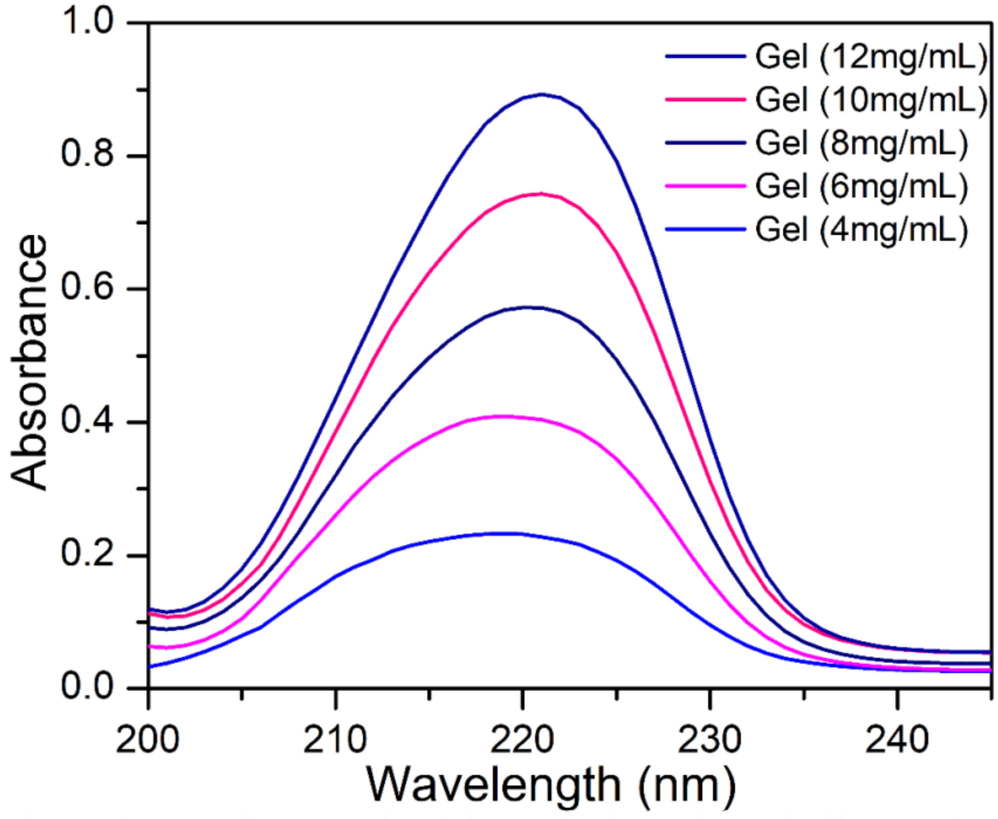

Figure S4. UV-Vis spectra of the ZW/GDL hydrogels with different concentrations.

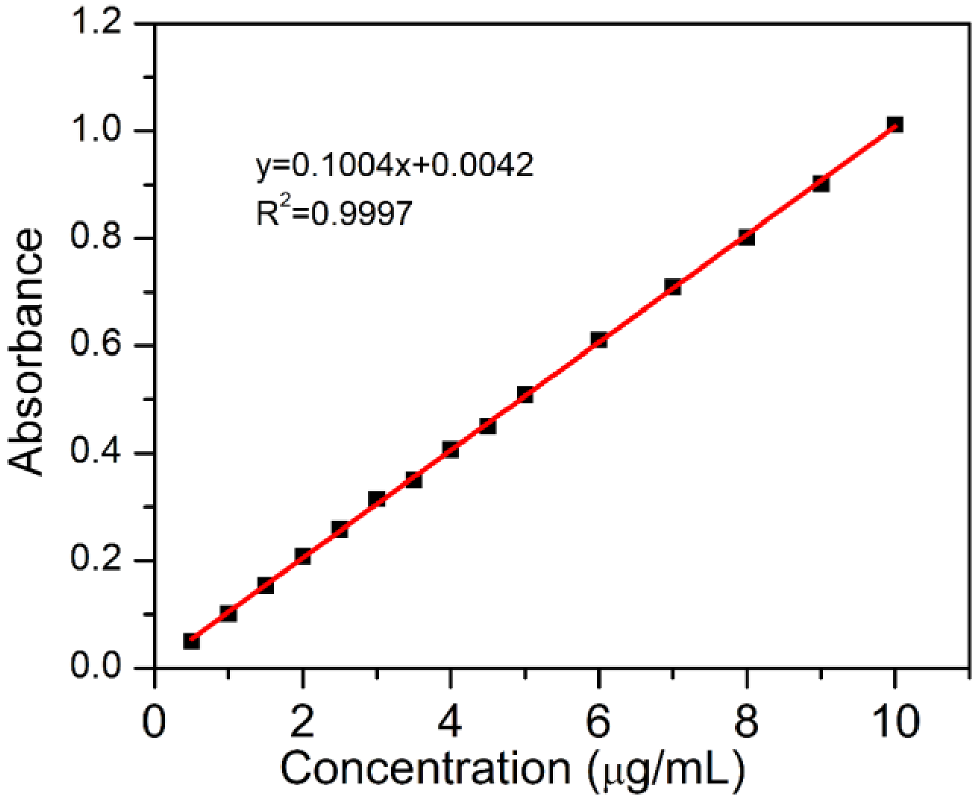

Figure S5. The standard curve of ZW/GDL hydrogel incubated with PBS solution (pH 7.4) at 37 °C.

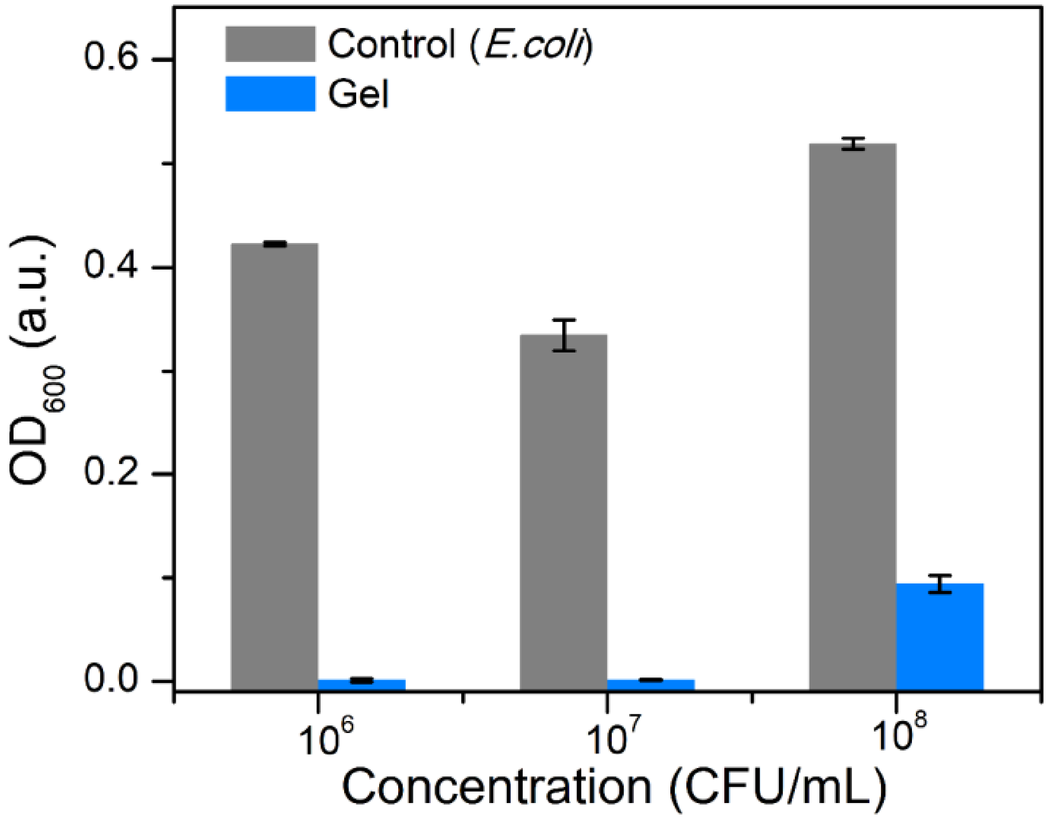

Figure S6. Concentration-dependent antimicrobial effect of the ZW/GDL hydrogel toward E.coli. bacteria.

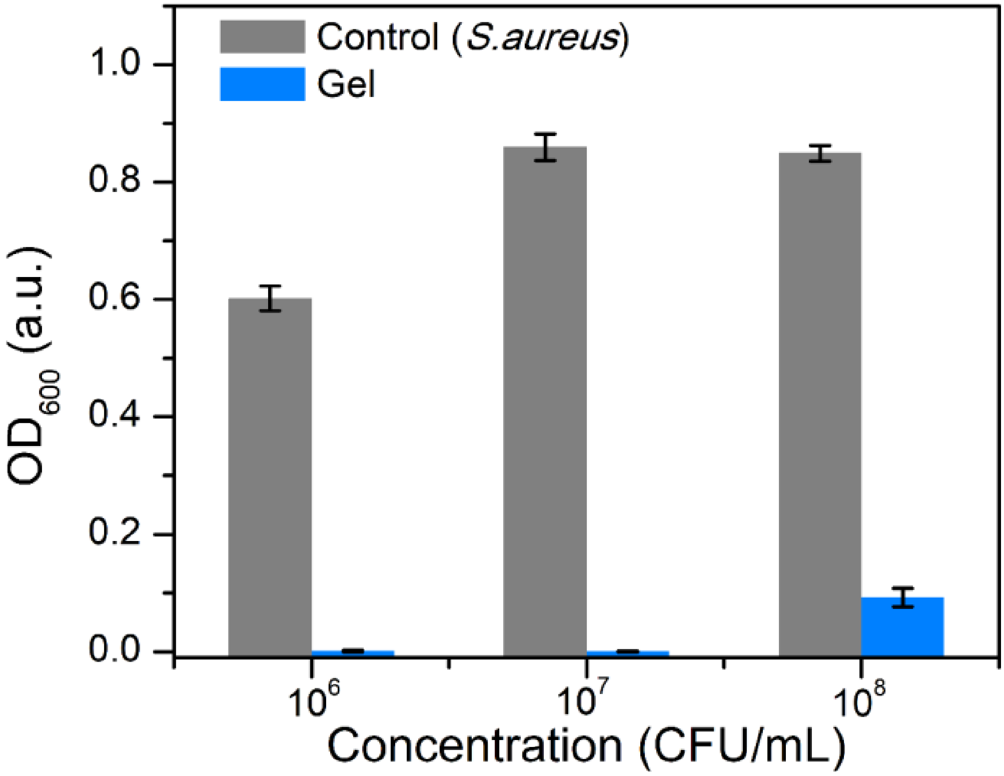

Figure S7. Concentration-dependent antimicrobial effect of the **ZW/GDL** hydrogel toward S. aureus bacteria.
